# Comparison of suture-button and screw fixation in the treatment of ankle syndesmotic injuries: Cohort study protocol

**DOI:** 10.1097/MD.0000000000021679

**Published:** 2020-08-07

**Authors:** Xiaoning Liu, Guang Jin, Chengdong Piao, Zhuan Zhong, Fei Chang, Bingzhe Huang

**Affiliations:** a Orthopaedic Medical Center, The 2nd Hospital of Jilin University; b Department of Orthopaedic, The First Hospital of Jilin University, Jilin, China.

**Keywords:** Ankle syndesmotic injuries, suture-button, syndesmotic screw, protocol

## Abstract

**Background:**

Syndesmotic injuries account for a significant number of ankle injuries. There is no consensus regarding the recommended method of treatment. The purpose of this study was to evaluate:

(1) functional outcomes,
(2) implant survivorship,
(3) complications, and
(4) radiographic analysis in patients who underwent fixtion with either syndesmotic screw or suture-button technique by the same experienced surgeon.

**Methods:**

This study was performed and reported in accordance with the Strengthening the Reporting of Observational studies in Epidemiology checklist. The records of 200 patients with ankle fractures who had undergone surgical treatment in our clinics between January 2014 and January 2018 were retrospectively investigated. This retrospective cohort study was approved by the institutional review board in the 2nd Hospital of Jilin University. The primary outcome measure was the American Orthopaedic Foot and Ankle Society ankle-hindfoot scale and the Foot Function Index. Secondary outcome measures included visual analog scale score, complications, range of movement of ankle, reoperations, and radiologic outcomes. For statistical comparison of the clinical and radiologic findings between the 2 groups, we used SPSS, version 21.0 (SPSS, Chicago, IL), statistical software. *P* Values of < .05 were considered statistically significant.

**Conclusion:**

The hypothesis was that the SB technique would achieve better functional outcomes as compared to the syndesmotic screw technique after surgery.

**Trial registration:**

This study protocol was registered in Research Registry (researchregistry5793).

## 1 Introduction

Syndesmotic injuries account for a significant number of ankle injuries, especially in athletic patient populations, with observed incidences as high as 25% in certain sportspecific cohorts.^[1]^ The treatment of such injuries ranges from nonoperative management of mild injuries to allograft reconstruction for injuries that result in chronic pain and instability.^[2–7]^ However, the current standard operative practice achieves reduction of the syndesmosis via proximally placed transosseous fixation devices, most commonly using syndesmotic screw (SS) or suture-button (SB) constructs.^[8]^

SS is the conventional approach to syndesmotic stabilization. Screw sizes vary from 3.5 mm to 6.0 mm and can involve either 3 or 4 cortical fixation. Screw fixation may be associated with complications including non-anatomic reduction, metalware irritation, broken and loose screws and limited range of motion.^[9,10]^ SB technique was developed to address some concerns of the SS technique; potential advantages include allowing physiological movement of the syndesmosis, anatomic healing, the ability to commence earlier rehabilitation, and typically avoiding implant removal.^[11–13]^ Several randomized controlled trials recently showed that the SB technique resulted in similar clinical outcomes in addition to lower rates of malreduction and complications as compared with the SS technique, albeit at higher cost.^[14–16]^ Despite several studies examining the benefits and disadvantages of both treatments, there is no consensus regarding the recommended method of treatment.^[17,18]^

Due to a lack of direct comparison between the clinical outcomes of these 2 techniques in current literature, uncertainty remains regarding the superiority of either method. The purpose of this study was to evaluate:

(1) functional outcomes,
(2) implant survivorship,
(3) complications, and
(4) radiographic analysis in patients who underwent fixtion with either SS or SB technique by the same experienced surgeon.

The hypothesis was that the SB technique would achieve better functional outcomes as compared to the SS technique after surgery.

## 2 Materials and methods

### 2.1 Study design and population

This study was performed and reported in accordance with the Strengthening the Reporting of Observational studies in Epidemiology checklist. The records of 200 patients with ankle fractures who had undergone surgical treatment in our clinics between January 2014 and January 2018 were retrospectively investigated. This retrospective cohort study was approved by the institutional review board in the 2nd Hospital of Jilin University (202007) and was registered in the research registry (researchregistry5793). Patients aged 18 years to 70 years who had suffered an acute syndesmotic injury, with or without an OTA/AO type 44 C ankle fracture, were assessed for study inclusion. Exclusion criteria were polytrauma, open fracture, inability to consent, symptomatic ankle osteoarthritis, neurologic impairment of the lower extremities, and present or previous injury to the lower extremities that could impair rehabilitation.

### 2.2 Techniques

All procedures were performed with patients in the supine position under general or spinal anesthesia with antibiotic prophylaxis and tourniquet control. In the SS group, standard ankle orthosis techniques were used to internally fix fractures of the fibula or tibia requiring fixation, and a large ankle orthosis clamp with the ankle in neutral position was used to reduce and maintain syndesmosis. Reduction was confirmed under image intensifier. The patients in the SS group were treated with a fully threaded, self-tapping, 4.5-mm cortical SS. A 3.2-mm hole was drilled through 4 cortices just proximal to the tibiofibular joint, with the ankle in neutral position. In the SB group, the needle attached to the leading oblong Button was passed through the holes and out from the intact medial skin along with the pull-through sutures. Once the medial button was passed through the medial tibial cortex, the assembly was tensioned by pulling the free ends of the FiberWire on the lateral side. Once both the buttons were seated flush with the bone, the free ends of the FiberWire were hand tied on the lateral side and cut 1 cm long and buried.

### 2.3 Postoperative treatment

The postoperative treatment protocol was similar in both groups. The ankle was immobilised in a below-the-knee cast with the ankle joint at a 90° for 6 weeks with partial weight bearing. At 6 weeks, the cast was removed, the ankle was examined, and a research physiotherapist instructed the patient in rehabilitation exercises. No additional bracing was used and weight bearing was allowed as tolerated. Patients visited the outpatient clinic at 2, 6, and 12 weeks. Joint congruity and fracture healing were assessed at each time via plain radiographs. Additional visits were scheduled if necessary. SS was removed only if local irritation occurred.

### 2.4 Outcome variables and measurements

All patients had a minimum of 1-year follow-up after surgery, with serial clinical examinations and radiographs. Patients were examined at 3 weeks, 6 weeks, and 3, 6, and 12 months postoperatively. The primary outcome measure was the American Orthopaedic Foot and Ankle Society (AOFAS) ankle-hindfoot scale and the Foot Function Index (FFI). The AOFAS and FFI scale were subdivided into subjective and objective categories scored together. Both AOFAS and FFI scores range from 0 to 100, with higher scores indicating better function. Secondary outcome measures included visual analog scale score, complications, range of movement of ankle, reoperations, and radiologic outcomes.

### 2.5 Statistical analysis

For statistical comparison of the clinical and radiologic findings between the 2 groups, we used SPSS, version 21.0 (SPSS, Chicago, IL), statistical software. Fisher exact test was used to analyze the gender differences between the 2 groups. For the outcomes followed a normal distribution, we would conduct statistical comparisons using the independent *t* test. For the outcomes did not follow a normal distribution, we would use the nonparametric Mann-Whitney *U* test. *P* Values of <.05 were considered statistically significant.

## 3 Result

The results will be shown in Table 1.

**Table 1:**
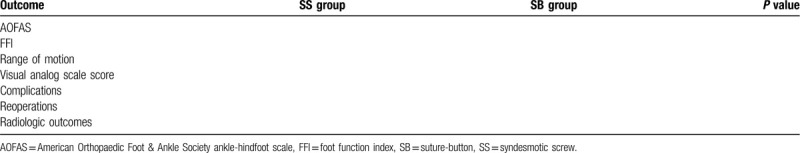
Postoperative outcomes.

## 4 Discussion

The purpose of this study was to evaluate:

(1) functional outcomes,
(2) implant survivorship,
(3) complications, and
(4) radiographic analysis in patients who underwent fixtion with either SS or SB technique by the same experienced surgeon.

The hypothesis was that the SB technique would achieve better functional outcomes as compared to the SS technique after surgery. The limitations of our study included those inherent in any retrospective cohort study, including the possibility of selection or observational bias. This study also did not address long-term follow-up (10 years) as our study relied on electronic medical records kept since 2014.

## Author contributions

Xiaoning Liu planned the study design and wrote the study protocol. Bingzhe Huang, and Chengdong Piao reviewed the study protocol. Xiaoning Liu, Guang Jin, and Fei Chang will recruit participants and collect data. All of the authors have read, commented on, and contributed to the submitted manuscript.
